# Stopping use of E-cigarettes and smoking combustible cigarettes: findings from a large longitudinal digital smoking cessation intervention study in the United States

**DOI:** 10.1186/s13104-024-06939-w

**Published:** 2024-09-27

**Authors:** Donghee N. Lee, Jamie M. Faro, Elise M. Stevens, Lori Pbert, Chengwu Yang, Rajani S. Sadasivam

**Affiliations:** 1 Department of Population and Quantitative Health Sciences, Division of Preventive and Behavioral Medicine, UMass Chan Medical School, 368 Plantation Street, Worcester, MA USA 01605; 2 Department of Population and Quantitative Health Sciences, Division of Health Informatics and Implementation Science, UMass Chan Medical School, 368 Plantation Street, Worcester, MA USA 01605; 3 Department of Population and Quantitative Health Sciences, Division of Biostatistics and Health Services Research, Measurement and Outcome Section, Department of Obstetrics and Gynecology, UMass Chan Medical School, 368 Plantation St., Worcester, MA USA 01605

**Keywords:** Smoking Cessation, Digital intervention, Electronic cigarettes, Cessation Aid, Smoking characteristics

## Abstract

**Objective:**

Digital interventions have been widely implemented to promote tobacco cessation. However, implementations of these interventions have not yet considered how participants’ e-cigarette use may influence their quitting outcomes. We explored the association of e-cigarette use and quitting smoking within the context of a study testing a digital tobacco cessation intervention among individuals in the United States who were 18 years and older, smoked combustible cigarettes, and enrolled in the intervention between August 2017 and March 2019.

**Results:**

We identified four e-cigarette user groups (*n* = 990) based on the participants’ baseline and six-month e-cigarette use (non-users, *n* = 621; recently started users, *n* = 60; sustained users, *n* = 187; recently stopped users, *n* = 122). A multiple logistic regression was used to estimate the adjusted odds ratios (AOR) of six-month quit outcome and the e-cigarette user groups. Compared to e-cigarette non-users, the odds of quitting smoking were significantly higher among recently stopped users (AOR = 1.68, 95% CI [1.06, 2.67], *p* = 0.03). Participants who were most successful at quitting combustible cigarettes also stopped using e-cigarettes at follow-up, although many sustained using both products. Findings suggest that digital tobacco cessation interventions may carefully consider how to promote e-cigarette use cessation among participants who successfully quit smoking.

**Trial registration:**

ClinicalTrials.gov identifier NCT03224520 (July 21, 2017).

**Supplementary Information:**

The online version contains supplementary material available at 10.1186/s13104-024-06939-w.

## Introduction

Smoking is a leading cause of preventable death, disability, and serious illnesses worldwide [1–4]. Studies have shown that digital interventions can promote smoking cessation (e.g., web-based, mobile phone text messaging) [5–7]. Real-world programs have adopted these interventions, including as an adjunct to Quitlines [8] or as a standalone program (smokefree.gov) [9].

Individuals who smoke have used electronic cigarettes (e-cigarettes) to quit [10], and the medical and public health community has increasingly accepted the harm reduction benefits of e-cigarettes [11–14]. Evidence on the effectiveness of adults’ e-cigarette use on their smoking cessation efforts is mixed [10–14]. A review of clinical trials demonstrated that substituting combustible cigarettes with e-cigarettes has increased smoking quit rates compared to nicotine replacement therapy (NRT) or e-cigarettes without nicotine [15]. A national U.S. cohort study revealed that adults who used e-cigarettes on their own were less successful in quitting or preventing relapse [10]. To our knowledge, no study has explored how e-cigarette use would influence quit outcomes among adult participants of a digital smoking cessation intervention.

Our paper describes a secondary analysis of a large randomized controlled trial for a digital smoking cessation intervention (Smoker-to-Smoker (S2S)). We examined the association of e-cigarette use and quitting smoking among U.S. adults who participated in a six-month digital smoking cessation intervention. We explored: (1) the demographic characteristics of e-cigarette users, (2) the smoking characteristics of e-cigarette users, and (3) was e-cigarette use associated with quitting smoking? Our results have implications for the design of digital interventions in the context of increasing e-cigarette use.

## Methods

### Study design, setting, and participants

The study was approved by the UMass Chan Medical School’s Institutional Review Board (H00012329) and informed consent was obtained from each participant in accordance with the Declaration of Helsinki.

We examined a cohort of adults who participated in the S2S digital smoking cessation intervention [16] between August 2017 and March 2019 (funded by Patient-Centered Outcomes Research Institute (PCORI; award: CDR-1603-34645). Eligibility for the S2S trial included: (1) speaking English, (2) currently smoking (as determined by a self-report question, “Do you currently smoke?”), and (3) aged ≥ 18 years. The research protocol and main outcomes have been published [16, 17]. The total analytic sample for the current study was *n* = 990 after only including participants who self-reported their e-cigarette use status at baseline.

### The original smoker-to-smoker (S2S) intervention

In the S2S trial, participants were randomly assigned to the intervention (machine-learning recommender messaging, which incorporated participants’ feedback to improve the message selection in addition to their baseline readiness to quit) or comparison (standard motivational messaging, which only incorporated participants’ baseline readiness to quit) group and received smoking cessation messages that were selected from the same messaging database [18]. These messages were emailed for six months post-registration in the S2S trial for the same frequency (four messages in the first two weeks followed by two messages each week). The messages exclusively discussed combustible cigarettes and did not include information about e-cigarettes. Data were collected using an online survey form at baseline and at six months [19] (See “Additional file 2”).

### Data collection and measures

At baseline, we collected (1) demographic data: age, gender, race, ethnicity, education level, and perceived difficulty of accessing medical care, and (2) smoking characteristics: the number of cigarettes smoked per day, readiness to quit, and living with others who smoke. At six-months, quit outcome was assessed using the following question: “Do you currently smoke cigarettes (smoked even 1 puff in the last 7 days)?” with answer choices of yes or no [20].

E-cigarette use was assessed using one question: “How many days have you used an e-cigarette within the past 30 days?” with answer choices of “every day,” “some days,” “not at all,” “don’t know/not sure” [21] at baseline (assessed at one week) and follow-up (assessed at six months). We also collected data on participants’ reasons for using e-cigarettes: “Why did you use an e-cigarette?” with a single-choice answers of “every day to quit smoking,” “some days to cut down on my smoking,” “to use in places where I was not allowed to smoke cigarettes,” and “others.” [21] (For the survey questions, see “Additional file 1”).

### Statistical analysis

Four e-cigarette user groups were created based on participants’ response to the baseline and at follow-up e-cigarette use question: non-users (not at all at baseline, not at all at follow-up), recently started users (not at all at baseline, every day/some days at follow-up), sustained users (every day/some days at baseline, every day/some days at follow-up), and recently stopped users (every day/some days at baseline, not at all at follow-up). Some of these users had missing e-cigarette values at follow-up. We treated the follow-up missing values for e-cigarette use as continuing the baseline e-cigarette use status. These users with missing follow-up values were all assigned to the non-users and sustained groups only, as by definition, the recently stopped and recently started group had to have e-cigarette values at baseline and follow-up. We performed chi-square tests to compare the baseline measures of demographic and smoking characteristics for three e-cigarette user groups with those of the non-user group (reference group).

We applied multiple logistic regression to estimate the adjusted odds ratios (AOR) of smoking cessation associated with e-cigarette user groups. In this analysis, the dependent variable was quit smoking (point prevalence smoking cessation at 6 months). Consistent with the smoking cessation literature and statistically significant baseline characteristics, we controlled for demographics (age, gender, race/ethnicity, education, and perceived financial difficulty) and random assignment. We reported both completed cases and penalized imputation where we assigned missing values for quitting smoking outcome as smoking. We used SPSS v28 [22] for all analyses.

## Results

Of 990 participants, 31.2% (*n* = 309) reported using e-cigarettes every day or some days at baseline, while 68.8% (*n* = 681) reported not using them at baseline. Our six-month follow-up survey completion rate was 66.7%. Of those who completed the follow-up survey, 24.3% of the participants (*n* = 157) reported using e-cigarettes every day or some days, and 75.0% (*n* = 484) reported not using them at follow-up. Four groups were identified: non-users (*n* = 621; *n* = 242 missing), recently started users (*n* = 60), sustained users (*n* = 187; *n* = 88 missing), and recently stopped users (*n* = 122).

### Demographic and smoking characteristics

Table 1 presents the baseline measures of demographic characteristics of the four e-cigarette user groups (*n* = 990). Differences in age were statistically significant across the e-cigarette user groups (*p* < 0.001). Compared to e-cigarette non-users (3.9%, *n* = 24), a higher proportion of recently started users (10.0%, *n* = 6, *p* = 0.04), sustained users (16.0%, *n* = 30, *p* < 0.001), and recently stopped users (14.8%, *n* = 18, *p* < 0.001) were younger (19–24 years). Differences in race were statistically significant across the e-cigarette user groups (*p* = 0.035). Compared to non-users (14.7%, *n* = 89), a higher proportion of recently stopped users identified as African American (20.4%, *n* = 23, *p* = 0.04). Gender, ethnicity, education, and perceived financial difficulty of accessing medical care were not statistically different across e-cigarette user groups.

**Table 1:** Comparison of baseline demographic characteristics across e-cigarette user groups

Participant characteristics	Non-users^a^	Recently Started Users	Sustained Users	Recently Stopped Users
	621 (62.7%)	60 (6.1%)	187 (19.1%)	122 (12.1%)
Age***				
19–24	24 (3.9%)	6 (10.0%)*	30 (16.0%)***	18 (14.8%)***
25–34	108 (17.4%)	15 (25.0%)	54 (28.9%)	38 (31.1%)
35–44	110 (17.7%)	9 (15.0%)	37 (19.8%)	38 (31.1%)
45–54	99 (15.9%)	13 (21.7%)	20 (10.7%)	10 (8.2%)
55–64	213 (34.3%)	14 (23.3%)	34 (18.2%)	14 (11.5%)
65+	67 (10.8%)	3 (5.0%)	12 (6.4%)	4 (3.3%)
**Gender**				
Female	475 (76.5%)	48 (80.0%)	127 (67.9%)	87 (71.3%)
Male	146 (23.5%)	12 (20.0%)	60 (32.1%)	35 (28.7%)
Race*				
White	488 (80.8%)	48 (81.4%)	146 (84.4%)	80 (70.8%)*
African American	89 (14.7%)	5 (8.5%)	20 (11.6%)	23 (20.4%)
Other race^#^	27 (4.5%)	6 (10.2%)	7 (4.0%)	10 (8.8%)
**Ethnicity**				
Not Hispanic/Latino	549 (93.1%)	53 (91.4%)	159 (91.4%)	106 (92.2%)
Hispanic	41 (6.9%)	5 (8.6%)	15 (8.6%)	9 (7.8%)
**Education**				
Never attended/some high school	29 (4.7%)	1 (1.7%)	17 (9.1%)	5 (4.2%)
High school graduate	153 (24.6%)	20 (33.9%)	43 (23.2%)	31 (25.8%)
Some college/technical school	270 (43.5%)	30 (50.8%)	74 (40.0%)	51 (42.5%)
College graduate	169 (27.2%)	8 (13.6%)	51 (27.6%)	33 (27.5%)
**How hard is it for you (and your family) to pay for medical care?** ^b^				
Hard	424 (68.3%)	48 (80.0%)	119 (63.6%)	88 (72.1%)
Other	183 (29.5%)	11 (18.3%)	60 (32.1%)	30 (24.6%)
Don’t know	14 (2.3%)	1 (1.7%)	8 (4.3%)	4 (3.3%)

*p < 0.05, **p < 0.01, ***p < 0.001

Note: Overall chi-square tests were statistically significant for age and race, but not significant for other demographic characteristics (gender, ethnicity, education, and perceived financial difficulty). *P*-values represent statistically significant differences between the left column (non-users) and each column (recently started users, sustained users, recently stopped users)

^a^ Indicates the reference group for comparison

^#^ Other race includes Asian, American Indian or Alaska Native, Native Hawaiian or Other Pacific Islander

^b^ Perceived difficulty of accessing medical care was collapsed into hard (very hard, hard, somewhat hard), not very hard, and don’t know

Table 2 presents the baseline measures of smoking characteristics of the four e-cigarette user groups (*n* = 990). Differences in the number of cigarettes smoked per day, readiness to quit smoking, and living with others who smoke cigarettes were not statistically different across the e-cigarette user groups. Differences in e-cigarette use reasons were statistically significant across the e-cigarette user groups (*p* < 0.001). Compared to non-users (35.7%, *n* = 136), a higher proportion of recently stopped users (61.2%, *n* = 74, *p* < 0.001) reported that they used e-cigarettes on some days to reduce smoking cigarettes. Compared to non-users (19.4%, *n* = 74), sustained users (31.0%, *n* = 57, *p* < 0.001) reported that they used e-cigarettes to replace smoking in the prohibited areas.

**Table 2:** Comparison of baseline smoking characteristics across e-cigarette user groups

Smoking characteristics	Non-users^a^ (*n* = 621)	Recently Started Users (*n* = 60)	Sustained Users (*n* = 187)	Recently Stopped Users (*n* = 122)
**Cigarettes smoked per day**				
0–10	199 (32.0%)	18 (30.0%)	65 (34.8%)	40 (32.8%)
> 10 and < = 20	294 (47.3%)	32 (53.3%)	81 (43.3%)	58 (47.5%)
> 20	128 (20.6%)	10 (16.7%)	41 (21.9%)	24 (19.7%)
**Readiness to quit**				
Not thinking of quitting	23 (3.7%)	3 (5.0%)	11 (5.9%)	3 (2.5%)
Thinking of quitting	361 (58.1%)	33 (55.0%)	110 (58.8%)	62 (50.8%)
Set a quit date	153 (24.6%)	16 (26.7%)	39 (20.9%)	36 (29.5%)
Quit today	40 (6.4%)	6 (10.0%)	13 (7.0%)	11 (9.0%)
E-cigarette use reasons***				
Every day to quit smoking	100 (26.2%)	8 (15.7%)*	32 (17.4%)***	21 (17.4%)***
Some day to cut down on smoking	136 (35.7%)	14 (27.5%)	84 (45.7%)	74 (61.2%)
To use in smoking prohibited areas	74 (19.4%)	19 (37.3%)	57 (31.0%)	20 (16.5%)
Others	71 (18.6%)	10 (19.6%)	11 (6.0%)	6 (5.0%)
**Does anyone else living in your home smoke cigarettes?**				
Yes	248 (39.9%)	26 (43.3%)	78 (41.7%)	59 (48.4%)
No	373 (60.1%)	34 (56.7%)	109 (58.3%)	62 (51.6%)

Note: Overall chi-square tests were not statistically significant for cigarettes smoked per day, readiness to quit, and living with others who smoke. *P*-values represent statistically significant differences between the left column (non-users) and each column (recently started users, sustained users, recently stopped users)

^a^ Indicates the reference group for comparison

### Smoking cessation outcomes

Table 3 presents the six-month follow-up quit outcome (yes vs. no) of the four e-cigarette user groups. Differences in the quit outcomes were statistically significant across the e-cigarette user groups (*p* < 0.001). Compared to non-users (35.6%, *n* = 135), a higher proportion of recently stopped users reported quitting smoking at follow-up (56.6%, *n* = 69, *p* < 0.001).

**Table 3:** Comparison of six-month quitting smoking across e-cigarette user groups

	Non-users^a^ (*n* = 621)	Recently Started Users (*n* = 60)	Sustained Users (*n* = 187)	Recently Stopped Users (*n* = 122)
**Quit smoking** ^b^ ***				
Complete Cases *n*/*N* (%)				
Yes	135 (35.6%)	15 (25.0%)	40 (40.4%)	69 (56.6%)***
No	244 (64.4%)	45 (75.0%)	59 (59.6%)	53 (43.4%)
Missing = Smoking ^c^				
*n*/*N* (%)	242 (39.0%)	0 (0.0%)	88 (46.6%)	(0.0%)

Notes: The percentages of quit smoking responses were reported based on the total complete cases. Overall chi-square test results of the e-cigarette user group and quit smoking were statistically significant. *P*-values represent statistically significant differences between the left column (non-users) and each column (recently started users, sustained users, and recently stopped users). Due to attrition, quit outcomes of *n* = 330 participants are missing (completion rate at the follow-up was 66.7%)

^a^ Indicates the reference group for comparison

^b^ Quit smoking was assessed by reverse coding responses to the question on “Do you currently smoke cigarettes?”

^c^ Indicates missing data for six-month quit outcomes due to sample attrition. *p < 0.05, **p < 0.01, ***p < 0.001

**Fig. 1:**
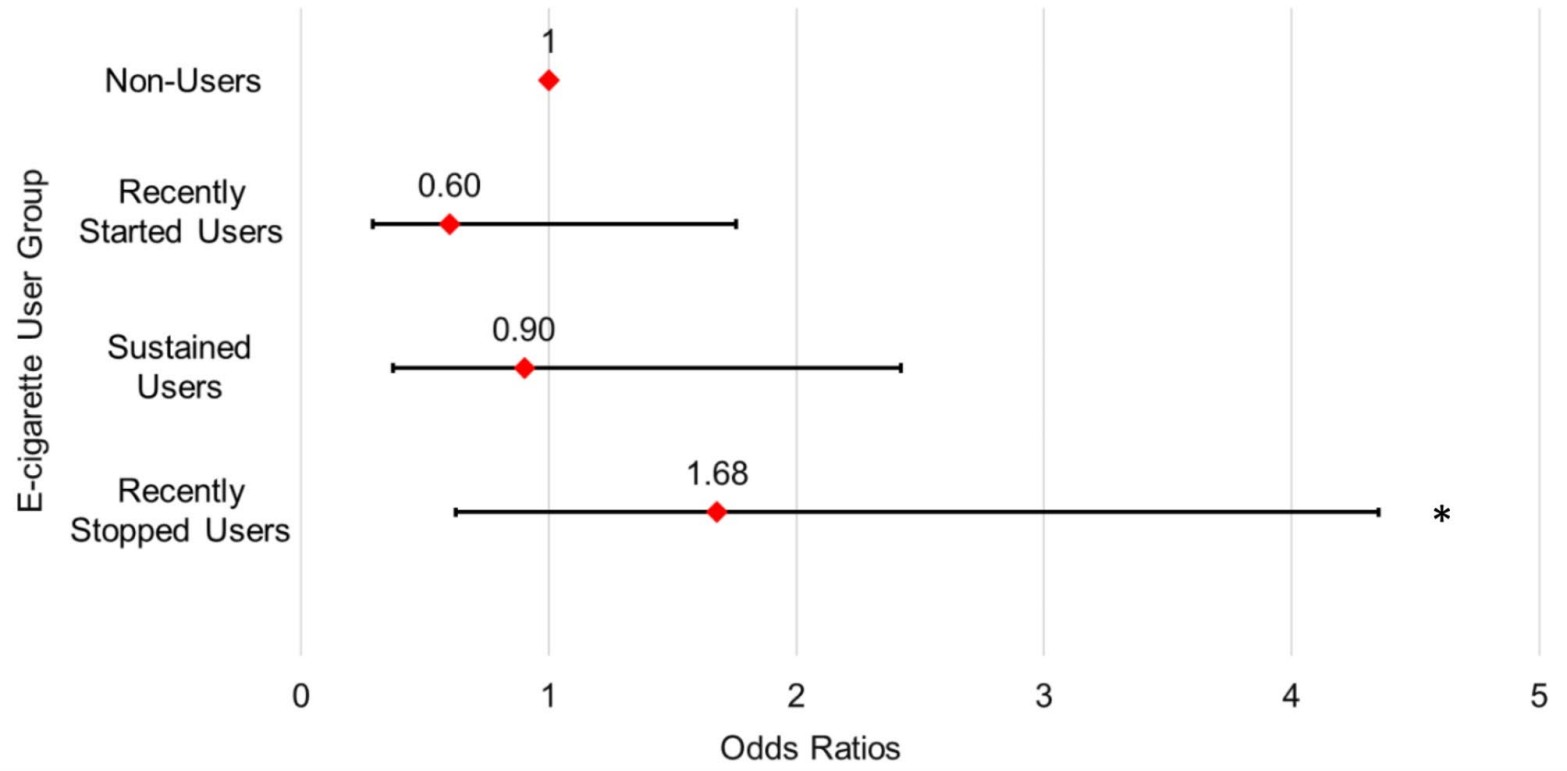
Adjusted model estimating association of quitting smoking and e-cigarette user groups

Figure 1 presents the adjusted odds ratio (AOR) of the six-month follow-up quit outcome by e-cigarette user groups. Compared to non-users, the odds of quitting smoking were significantly higher among recently stopped users (AOR = 1.68, 95% CI [1.06, 2.67], *p* = 0.03).

## Discussion

We examined the association of e-cigarette use and quitting smoking among participants of a six-month digital smoking cessation intervention. Participants’ demographic characteristics differed across e-cigarette user groups. More participants who used e-cigarettes both at baseline and follow-up were younger (19–24 years old) than those who did not use e-cigarettes at all. More participants who used e-cigarettes at baseline but stopped at follow-up identified as African Americans than those who did not use e-cigarettes at all. Overall, 56.6% of participants who stopped using e-cigarettes at follow-up also quit smoking.

Younger participants’ e-cigarette use indicates their possible e-cigarette exposure from their peers [23], marketing influence [24], or their lack of awareness of the health harms and addictiveness of e-cigarettes [25, 26]. This is concerning for those between 19 and 24 years who use e-cigarettes, as nicotine in e-cigarettes can harm their brain development [27]. Furthermore, young adults who used e-cigarettes at follow-up without successfully quitting smoking engaged in dual use of cigarettes and e-cigarettes. This is concerning, as dual use can pose greater health risk than exclusively using combustible cigarettes [28]. Thus, more intervention work is needed to help young adults quit using both e-cigarettes [29] and cigarettes. Additionally, more participants who stopped using e-cigarettes at follow-up identified as African American than white (20.4%), which differ from other findings that African American participants had higher e-cigarette use rates compared to their white counterpart [30], despite their generally lower overall e-cigarette use [31, 32].

Participants who initially used e-cigarettes but stopped at follow-up were more successful in quitting smoking than those who did not use e-cigarettes at all during the intervention. Differences in participants’ e-cigarette use reasons may explain this difference. More participants who recently stopped using e-cigarettes were more likely to report using e-cigarettes on some days to reduce smoking, whereas more participants who initiated or sustained using e-cigarettes reported using e-cigarettes in smokefree areas. These findings raise questions about the role of e-cigarette use in quit outcomes in the context of a digital smoking cessation intervention, as it may potentially lead to dual use [33], suggesting the need to examine challenges and motivations of those who use both products when designing interventions targeting this group. Prior randomized controlled trials that provided and encouraged e-cigarette use have shown that e-cigarettes can be used as a smoking cessation aid [15, 34]. However, many participants of these trials continued using e-cigarettes after quitting smoking. While we did not include messages about e-cigarettes in our study, digital smoking cessation interventions may also consider including messages promoting quitting both e-cigarettes and cigarettes. Research needs to identify the appropriate timing to discuss e-cigarettes as a cigarette substitution for harm reduction and eventually to quit e-cigarettes.

Overall, our study provides insights into how e-cigarette use may affect quitting smoking among adult participants of a digital smoking cessation intervention. It is possible that individuals who stopped using e-cigarettes at the end of the intervention had greater motivation and efficacy to adopt a healthier lifestyle, as has been shown in other trials [35, 36]. However, this conclusion requires a further investigation into how individuals’ e-cigarette use may interact with their stages of change in smoking to influence their quit outcomes. Therefore, careful consideration of how to promote both smoking and e-cigarette cessation may help improve effectiveness of future digital tobacco cessation interventions.

### Limitations

We are unable to make a causal association between e-cigarette use and participants’ quit outcomes, as e-cigarette use was not part of our intervention. Specific information about e-cigarettes (types, flavors, intensity of use), or intermediate e-cigarette use outcomes, or cigarette dependence measures were not collected, although more information on e-cigarette and cigarette use could provide more insights. The main outcome was self-reported smoking status. Future analysis should incorporate biochemical measures [37], to reduce the potential reporting bias. Additionally, our findings have limited statistical power from the small sample size. We did not adjust for multiple comparisons, as it may lead to false negative findings and reduce statistical power [38–40], thus not recommended for exploratory studies. Finally, our findings may not fully apply to the current tobacco marketplace, as the e-cigarette landscape has evolved (e.g., emergence of novel product types and regulations) since our data collection.

## Electronic supplementary material

Below is the link to the electronic supplementary material.

Supplementary Material 1

Supplementary Material 2

## Data Availability

Data are available upon request (PI: Sadasivam) at rajani.sadasivam@umassmed.edu.

## Abbreviations

S2S: Smoker-to-smoker
