# Supplementary material for: Stopping use of E-cigarettes and smoking combustible cigarettes: findings from a large longitudinal digital smoking cessation intervention study in the United States
Source: BMC Res Notes. 2024 Sep 27;17:276. doi: 10.1186/s13104-024-06939-w (PMC11438106; doi:10.1186/s13104-024-06939-w)
Supplement: Supplementary file 1 — Supplementary Material 1 [file 13104_2024_6939_MOESM1_ESM.docx]

**Survey Questions**

**Instructions.** We encourage you to answer all questions. Please follow instructions where noted. Thank you for your participation!

**Registration/Baseline Survey**

How did you hear about Decide2Quit?

Facebook Ad

Google Ad

Research Match

Smokefree.gov

Friend or Family Member

What is your age?

19-24

25-34

35-44

45-54

55-64

65+

Are you…?

Male

Female

What RACE do you consider yourself to be? (Select all that apply)

Black of African American

White

Asian

American Indian or Alaska Native

Native Hawaiian or Other Pacific Islander

Don’t Know/Not Sure

Refused

Other

Do you consider yourself to be Hispanic or Latino, that is a person of Mexican, Puerto Rican, Cuban, South or Central American, or other Spanish culture or origin regardless of race?

Hispanic or Latino

Not Hispanic or Latino

Don’t Know/Not sure

Refused

5.   About how many cigarettes do you smoke per day? (only numbers)

6.   First name/nickname

7.   Your Telephone or Cell Phone Number:(Please provide your Telephone or Cell Phone number using this format XXX-XXX-XXXX):

8.   People often go through different steps when quitting smoking. Where are you in the process?

I’m not thinking about quitting

I set a quit date

I quit today

I’ve already quit

What is the HIGHEST GRADE OR YEAR OF SCHOOL you completed?

Never attended school or only attended kindergarten

Grades 1 through 8 (Elementary)

Grades 9 through 11 (Some high school)

Grade 12 or GED (High school graduate)

College 1 year to 3 years (Some college or technical school)

College 4 years or more (College graduate)

How hard is it for you (and your family) to pay for medical care? Would you say it is (select one)

Very hard

Hard

Somewhat hard

Not very hard

Don't know

**One-Week Survey**

| What is the HIGHEST GRADE OR YEAR OF SCHOOL you completed? |
| --- |
| 1. Never attended school or only attended kindergarten 2. Grades 1 through 8 (Elementary) 3. Grades 9 through 11 (Some high school) 4. Grade 12 or GED (High school graduate) 5. College 1 year to 3 years (Some college or technical school) 6. College 4 years or more (College graduate) |
| How hard is it for you (and your family) to pay for medical care?  Would you say it is:  *(select one)* |
| 1. Very hard 2. Hard 3. Somewhat hard 4. Not very hard 5. Don't know |
| Have you ever visited a smoking cessation website before? |
| 1. Yes   1. No |
| Have you/Are you currently using any of the following *(check all that apply)*: |
| Nicotine Patches (Transdermal nicotine system)  Nicotine Nasal Spray  Nicotine Inhaler  Nicotine Lozenge  Nicotine Gum (Nicotine Polacrilex)  Chantix  Wellbutrin |
| Have you used/ever participated in tobacco counseling before? |
| 1. No 2. In person 3. Group 4. Telephone (1-800 Quitlines) 5. Internet |
| How soon after you wake up do you smoke your first cigarette? (Nicotine Dependence (Fagerstrom Test for Nicotine Dependence Scale) |
| 1. Within 5 minutes 2. 6-30 minutes 3. 31-60 minutes 4. After 60 minutes |
| Have you ever tried an e-cigarette, even just one time? |
| 1. Yes 2. No |
| How many days have you used an e-cigarette in the past 30 days? |
| 1. Every day 2. Some days 3. Not at all |
| Why did you use an e-cigarette? |
| 1. Every day to quit smoking 2. Some days to cut down on my smoking 3. To use in places where I was not allowed to smoke cigarettes 4. Other 5. If other, please elaborate: |
| Besides yourself, does anyone who lives in your home smoke cigarettes now? |
| 1. Yes 2. No |
| Would you be interested in a program that also includes your partner/and or spouse in a smoking cessation intervention |
| 1. Yes  0. No |
| Thinking about people you know who smoke cigarettes, how many are |
| 1. Immediate family 2. Extended family 3. Close friends, with whom you feel at ease and discuss private matters 4. Friends, with whom you feel at ease but DO NOT discuss private matters 5. Acquaintances, to whom you may say "hello" but about whom you know little 6. Co-workers |

**One Month Follow-up Survey**

{QUESTIONS #1 TO #9 WILL ONLY BE ASKED OF THE PEER RECRUITMENT ARM}

1. How would you best describe the nature of your relationship with the individual who referred you:

0 Your spouse or partner

1 Immediate family members (other than your spouse)

2 Extended family members and/or relatives

3 Close friend (a person you feel at ease with, can talk to about private matters)

4 Friend (someone you know personally, like, & trust but whom you do not share private matters)

5 Acquaintance (someone who you may say “hello” to, but know little about)

6 Co-Worker

2. How many other smokers have you told about our website, [www.decide2quit.org](http://www.decide2quit.com) ? ________

3. How many smokers did you attempt to refer to our website, [www.decide2quit.org](http://www.decide2quit.com) ?_________ (enter 0 if you haven’t referred anyone to our site) 🡪 if this is 0 SKIP TO #6 and language of questions will change based on no referrals

4. Which modes of communication did you use to refer other smokers? (please check all that apply)

1. Face-to-face
2. Telephone
3. Email
4. Facebook or a social networking site
5. Text message
6. The recruitment form on the decide2quit.org website

5. Of the smokers you tried to refer, what was the most common reason a smoker would refuse referral to the [www.decide2quit.org](http://www.decide2quit.com) website? (choose all that apply)

1. Not ready to quit smoking
2. No computer/internet access
3. Cannot use computer/website (lack of technical abilities)
4. No time
5. Other, please specify: _________________________

6. Please indicate your agreement with the statements below: (adaptation of STORIES SIS)

**Recruiting Other Smokers to Decide2Quit:**

|  | Strongly Agree | Agree | Neutral | Disagree | Strong Disagree |
| --- | --- | --- | --- | --- | --- |
| Was/ would be beneficial to my own quit smoking efforts. |  |  |  |  |  |
| Motivated/would motivate me to get support from those around me to quit smoking |  |  |  |  |  |
| Increased/would increase my craving for cigarettes. |  |  |  |  |  |
| Made/would make me feel like I was being helpful to my family and friends who are smokers |  |  |  |  |  |
| Was/ would be burdensome |  |  |  |  |  |

7. Please indicate your agreement with the statements below:

|  | Strong Agree | Agree | Neutral | Disagree | Strong Disagree | Not Applicable |
| --- | --- | --- | --- | --- | --- | --- |
| The referral tools provided at the www.decide2quit.org website were helpful in my referral efforts: |  |  |  |  |  |  |
| The instructions provided at the www.decide2quit.org website were clear and easy to follow: |  |  |  |  |  |  |

8. What do you think are the biggest challenges of recruiting friends or family members to a website like Decide2Quit.org? The challenges might be for yourself or for the people you recruit to the site. (please share 1-3 points)

9. What do you think are the biggest benefits of recruiting friends or family members to a website like Decide2Quit.org? The benefits might be for yourself or for people you recruit to the site. (please share 1-3 points)

{ALL STUDY PARTICIPANTS ARE REQUIRED TO RESPOND TO QUESTIONS #10 TO #13}

10. We would like to know how much the Quit Smoking Messaging System has influenced you, how much do you agree with the following statement.  *The Quit Smoking Messaging System has influenced me…*

|  | Strongly Agree | Agree | Neutral | Disagree | Strongly Disagree |
| --- | --- | --- | --- | --- | --- |
| Quit Smoking | 🞏 | 🞏 | 🞏 | 🞏 | 🞏 |
| Talk to a doctor about quitting smoking | 🞏 | 🞏 | 🞏 | 🞏 | 🞏 |
| Get support from those around you to help quit smoking | 🞏 | 🞏 | 🞏 | 🞏 | 🞏 |
| Set a quit date | 🞏 | 🞏 | 🞏 | 🞏 | 🞏 |
| Use Nicotine Replacement Therapy like the patch or gum | 🞏 | 🞏 | 🞏 | 🞏 | 🞏 |
| Make a list of reasons to quit smoking | 🞏 | 🞏 | 🞏 | 🞏 | 🞏 |
| Use behavioral strategies like distraction or substitution | 🞏 | 🞏 | 🞏 | 🞏 | 🞏 |

11. Thinking about your overall experience with the system, how much would you agree with the following statement…….

I would recommend the Quit Smoking Messaging System to my friends and family.

- Strongly Agree
- Agree
- Neutral
- Disagree
- Strongly Disagree

12. Thinking about the messages you received during the intervention, what were the things you liked about the message?

13. Thinking about the messages you received during the intervention, what additional things would you have liked to see in the messages?

14. What message or advice would you send to someone trying to quit smoking?

**6-Month Survey**

Date (MM/DD/YYYY): _____/______/_______

1. Do you currently smoke cigarettes (smoked even 1 puff in the last 7 days)?

- Yes
- No 🡪 ***skip next question***

1. About how many cigarettes do you smoke per day? __________
2. Have you quit or had any quit attempts since registering on the *Decide2Quit* website, about six months ago?

- Yes
- No 🡪 ***skip next question***

1. If Yes, how many quit attempts did you have? __________________________

b. When was your first quit attempt?

MONTH:

DAY:

YEAR:

1. How soon after you wake up do you smoke your first cigarette?
   - Within 5 minutes
   - 6-30 minutes
   - 31-60 minutes
   - After 60 minutes
2. Everyone is at a different stage in the quitting process, what is your current smoking status?
   - I am not thinking about quitting
   - I am thinking about quitting
   - I have set a quit date
   - I quit today
   - I have already quit
3. How many days have you used an e-cigarette within the past 30 days?

- Every day
- Some days
- Not at all 🡪 ***skip next questions***
- Don’t know/Not Sure 🡪 ***skip next questions***

1. Could you please tell us why you use e-cigarettes? (Please check one)

- Every day to quit smoking
- Some days to cut down on my smoking
- To use in places where I am not allowed to smoke cigarettes
- Other: please elaborate: _________________

1. We would like to know how much the **Decide2Quit study** has influenced you, how much do you agree with the following statement.

*The Decide2Quit study has influenced me to…...*

|  | Strongly Agree | Agree | Neutral | Disagree | Strongly Disagree |
| --- | --- | --- | --- | --- | --- |
| 1. Quit Smoking | 🞏 | 🞏 | 🞏 | 🞏 | 🞏 |
| 1. Talk to a doctor about quitting smoking | 🞏 | 🞏 | 🞏 | 🞏 | 🞏 |
| 1. Get support from those around you to help quit smoking | 🞏 | 🞏 | 🞏 | 🞏 | 🞏 |
| 1. Set a quit date | 🞏 | 🞏 | 🞏 | 🞏 | 🞏 |
| 1. Use Nicotine Replacement Therapy like the patch or gum | 🞏 | 🞏 | 🞏 | 🞏 | 🞏 |
| 1. Make a list of reasons to quit smoking | 🞏 | 🞏 | 🞏 | 🞏 | 🞏 |
| 1. Use behavioral strategies like distraction or substitution | 🞏 | 🞏 | 🞏 | 🞏 | 🞏 |

1. While participating in the **decide2quit** study, you had access to the decide2quit *messaging system*. Thinking about your overall experience, how much would you agree with the following statement(s)…

|  | Strongly Agree | Agree | Neutral | Disagree | Strongly Disagree |
| --- | --- | --- | --- | --- | --- |
| 1. I was mentally involved in reading the daily message. | 🞏 | 🞏 | 🞏 | 🞏 | 🞏 |
| 1. The messages affected me emotionally. | 🞏 | 🞏 | 🞏 | 🞏 | 🞏 |
| 1. The messages were relevant to my everyday life. | 🞏 | 🞏 | 🞏 | 🞏 | 🞏 |
| 1. I would recommend the Decide2Quit messaging system to my friends and family. | 🞏 | 🞏 | 🞏 | 🞏 | 🞏 |

1. Have you/Are you currently using any of the following (select all that apply)

- Nicotine Patch (Transdermal nicotine system)
- Nicotine Nasal Spray
- Nicotine Inhaler
- Nicotine Lozenge or Mini-Lozenge
- Nicotine Gum
- Chantix
- Wellbutrin

1. Within the last six months, have you ever used/ever participated in tobacco counseling? (select all that apply)

- Individual (In person)
- Group (In-person)
- Telephone (1-800 Quit lines)
- Internet
- Other: Please specify__________________________

{THE FOLLOWING QUESTIONS WILL ONLY BE ASKED OF THE PEER RECRUITMENT ARM}

1. How would you best describe the nature of your relationship with the individual who referred you:

- Your spouse or partner
- Immediate family members (other than your spouse)
- Extended family members and/or relatives
- Close friend (a person you feel at ease with, can talk to about private matters)
- Friend (someone you know personally, like, & trust but whom you do not share private matters)
- Acquaintance (someone who you may say “hello” to, but know little about)
- Co-Worker

1. How many other smokers have you refer or told about our website, [www.decide2quit.org](http://www.decide2quit.com) ?

*Please note that referrals can be made* ***face-to-face****,* ***via email, telephone, Facebook or any other social networking site***

________ (enter 0 if you have not referred or told anyone about our website)

If 0 🡪 ***Skip to question 19***

15. Which modes of communication did you use to refer other smokers? (please check all that apply)

- Face-to-face
- Telephone
- Email
- Facebook
- Other social networking site: Specify______________________
- Text message
- The recruitment form on the [www.decide2quit.org](http://www.decide2quit.com) website

16. Of the smokers you tried to refer, what was the most common reason a smoker declined your referral to the [www.decide2quit.org](http://www.decide2quit.com) website? (choose all that apply)

- Not ready to quit smoking
- No computer/internet access
- Cannot use computer/website (lack of technical abilities)
- Not interested
- Other, please specify: ________________________

17. Please indicate your agreement with the below statements:

Recruiting Other Smokers to [www.decide2quit.org](http://www.decide2quit.com) ………

|  | Strongly Agree | Agree | Neutral | Disagree | Strongly Disagree |
| --- | --- | --- | --- | --- | --- |
| 1. Was beneficial to my own quit smoking efforts. | 🞏 | 🞏 | 🞏 | 🞏 | 🞏 |
| 1. Motivated me to get support from those around me to quit smoking | 🞏 | 🞏 | 🞏 | 🞏 | 🞏 |
| 1. Increased my craving for cigarettes. | 🞏 | 🞏 | 🞏 | 🞏 | 🞏 |
| 1. Made me feel like I was being helpful to my family and friends who are smokers | 🞏 | 🞏 | 🞏 | 🞏 | 🞏 |
| 1. Was burdensome | 🞏 | 🞏 | 🞏 | 🞏 | 🞏 |

18. Please indicate your agreement with the below statement:

|  | Strongly Agree | Agree | Neutral | Disagree | Strongly Disagree |
| --- | --- | --- | --- | --- | --- |
| 1. The referral tools provided at the www.decide2quit.org website were helpful in my referral efforts: |  |  |  |  |  |
| 1. The instructions provided at the www.decide2quit.org website were clear and easy to follow: |  |  |  |  |  |

19. What do you think are the biggest challenges of recruiting friends or family members to a website like [www.decide2quit.org](http://www.decide2quit.com)? The challenges might be for yourself or for the people you recruit to the site. (please share 1-2 points)

20. What do you think are the biggest benefits of recruiting friends or family members to a website like [www.decide2quit.org](http://www.decide2quit.com)? The benefits might be for yourself or for people you recruit to the site. (please share 1-2 points)

21. For participants who answer, “I quit today” or “I have already quit” on question 4: Would you be willing to verify your quit status by taking a NicAlert® saliva test?

- Yes
- No 🡪 ***skip next question***

If you agree:

We will mail you the test, along with detailed instructions on how to administer the NicAlert@ saliva test which involves placing a piece of paper in your mouth to collect a saliva sample.

Afterward we ask you to take a picture and email or upload the image back to our servers. You will receive an **electronic $50-dollar amazon gift card in your email** once we receive your image

22. Thanks for agreeing to participate! To mail you the test package we need you to provide your mailing details below:

FIRST NAME:

LAST NAME

STREET ADDRESS:

CITY:

STATE:

ZIP CODE:

23. Would you be interested in participating in additional tobacco cessation studies in the future? [IF YOU ARE INTERESTED, WE MAY CONTACT YOU AS NEEDS ARISE.]

- Yes
- No

24. We need to get a little bit of information to email you your electronic amazon gift card.

Please verify your email address: ___________________________________

***Thank you for completing this survey!***

If you have any questions or concerns, please feel free to contact the study coordinator.
