# Supplementary material for: Stopping use of E-cigarettes and smoking combustible cigarettes: findings from a large longitudinal digital smoking cessation intervention study in the United States
Source: BMC Res Notes. 2024 Sep 27;17:276. doi: 10.1186/s13104-024-06939-w (PMC11438106; doi:10.1186/s13104-024-06939-w)
Supplement: Supplementary file 2 — Supplementary Material 2 [file 13104_2024_6939_MOESM2_ESM.docx]

**Supplementary File**

**Recruitment and Randomization**

**Recruitment.** Details of the S2S interventions have been published (Faro et al., 2019; Faro et al., 2021). Briefly, we recruited participants between August 2017 and March 2019 using multiple approaches: Peer recruitment (18.4%), Facebook Ads (34.0%), Google Ads (13.4%), ResearchMatch (23.9%), and Smokefree.gov (10.3%). The research team developed and posted online advertisements on Google and Facebook that were customized to appear to smokers who were using search terms related to quitting smoking. A summary of the research project was posted on SmokeFree.gov’s “Join a Research Study” webpage with the research staff’s contact information. We also contacted volunteers from ResearchMatch (a free and secure online tool developed by Vanderbilt University) if they matched our eligibility criteria and agreed to be contacted. Lastly, we used peer recruitment by sending encouragement emails to participants with a secure email form and a Facebook plugin to share with their friends and family. After study enrollment, data were collected using an online survey form at baseline and at six-month. We encouraged participants to complete the follow-up survey by sending them up to five emails and calling them on the phone up to six times. Additionally, participants could receive up to $100 in incentives for completing the surveys ($25 each for the one-week and one-month survey and $50 for the six-month survey).

**Randomization:** Once participants were recruited into the study, they were randomized into either a machine-learning recommender messaging or standard motivational messaging group. Participants in both groups received smoking cessation messages that were selected from the same messaging database.

**References**

Faro JM, Orvek EA, Blok AC, et al. Dissemination and Effectiveness of the Peer Marketing and Messaging of a Web-Assisted Tobacco Intervention: Protocol for a Hybrid Effectiveness Trial. *JMIR Res Protoc*. 2019;8(7):e14814. doi:10.2196/14814

Faro JM, Nagawa CS, Orvek EA, et al. Comparing recruitment strategies for a digital smoking cessation intervention: Technology-assisted peer recruitment, social media, ResearchMatch, and smokefree.gov. *Contemporary Clinical Trials*. 2021;103:106314. doi:10.1016/j.cct.2021.106314

**Supplementary Table-1.** Adjusted associations of e-cigarette user groups and the number of cigarettes used per day.

| E-cigarette User Group | B | SE | p-value | 95% CI |
| --- | --- | --- | --- | --- |
| Non-users | -0.48 | 0.87 | 0.58 | -2.179, 1.223 |
| Recently started users | 1.18 | 1.31 | 0.37 | -1.397, 3.765 |
| Sustained users | 0.59 | 1.22 | 0.96 | -2.330, 2.449 |
| Recently stopped users | -0.12 | 1.20 | 0.92 | -2.470, 2.223 |

Note: Unstandardized coefficients and standard error were reported. Models controlled for age, sex, race, ethnicity, education, perceived financial difficulty, and random assignment.

**Supplementary Table-2.** Adjusted associations using sensitivity analysis.

| E-cigarette User Group | AOR | p-value | 95% CI |
| --- | --- | --- | --- |
| Non-users (reference) |  |  |  |
| Recently started users | 1.57 | 0.337 | 0.625, 3.934 |
| Sustained users | 0.898 | 0.809 | 0.375, 2.148 |
| Recently stopped users | 1.253 | 0.580 | 0.564, 2.787 |

Note: Adjusted odds ratios were reported. Models controlled for age, sex, race, ethnicity, education, perceived financial difficulty, and random assignment. Sensitivity analysis with penalty imputation was performed, where missing values at follow-up were excluded from analysis.
